# A study on oral microbiome characteristics and differences in epilepsy adults with depressive symptoms based on 16S rRNA sequencing

**DOI:** 10.3389/fmicb.2026.1894254

**Published:** 2026-09-16

**Authors:** Xinyi Zhang, Yan Guo, Shoubin Gao, Songbai Li, Yiqiao Du, Dongjun Wan, Zhao Liu

**Affiliations:** 1 940th Hospital of Joint Logistics Support Force of PLA, Gansu, China; 2 The First Clinical Medical School, Gansu University of Chinese Medicine, Gansu, China; 3 The General Hospital of Western Theater Command PLA, Chengdu, China

**Keywords:** 16S rRNA sequencing, differential genera, epilepsy, epilepsy with depressive symptoms, oral microbiota, oral-brain axis

## Abstract

**Background:**

Depression is a common comorbidity in epilepsy, but its underlying mechanisms are poorly understood. Although the “oral-brain axis” has gained increasing attention in neuropsychiatric disorders, oral microbial characteristics in epileptic patients with depressive symptoms remain largely unexplored. This study aimed to compare the oral microbial composition between epileptic patients with and without depressive symptoms and to identify bacterial genera and functional pathways that may be associated with depressive symptoms.

**Methods:**

The study included 59 adult patients with epilepsy. Based on the Neuropsychiatric Depression Scale for Epilepsy (NDDI-E) scores, the participants were divided into two groups: patients with epilepsy with depressive symptoms (EDS, NDDI-E > 12, *n* = 28) and patients with epilepsy alone (EP, NDDI-E ≤ 12, *n* = 31). Tongue swab samples were collected and analyzed by 16S rRNA MiSeq sequencing. Firth-corrected logistic regression analysis was performed to identify potential factors associated with depressive symptoms in epilepsy.

**Results:**

No significant differences were found between the two groups in terms of general clinical data or oral microbial diversity (α- and β-diversity). Using an uncorrected *p* value < 0.05 as the screening criterion, microbial composition analysis revealed that *Dialister* and *Olsenella* were enriched in EDS, while *Bacillus*, *Fusicatibacter*, and *[Eubacterium]_coprostanoligenes_group* were enriched in EP. LEfSe analysis further suggested that *Dialister* and *Bacillus* were key differential genera with discriminatory capability. Based on PICRUSt2’s functional prediction results, the predicted functional abundance annotated to the RIG-I-like receptor signaling pathway was higher in the EDS group than in the EP group. Multivariate Firth-corrected logistic regression analysis showed that the abundance of *Dialister* remained associated with EDS (OR = 1.646, 95% CI: 1.007–2.896, *p* = 0.047), after adjusting for potential confounding factors.

**Conclusion:**

This study utilizing 16S rRNA sequencing elucidates alterations in the oral microbiome associated with epilepsy and concurrent depressive symptoms. Significantly, the enrichment of *Dialister* is independently correlated with depressive symptoms following adjustment for confounding variables, indicating its potential as a microbial biomarker. Functional prediction analysis further indicated that the associated oral microbial alterations may involve innate immune-related functional characteristics, particularly the RIG-I-like receptor signaling pathway. These findings provide new clues for further investigating the potential association between oral microbiota and epilepsy with depressive symptoms.

## 1 Introduction

Epilepsy is one of the most common chronic neurological disorders, affecting more than 70 million people worldwide (Gurgu et al., 2021). The prevalence of comorbid psychiatric disorders is as high as 43.3% (Gurgu et al., 2021), and that of comorbid depressive symptoms among adults can reach 32% (Rashid et al., 2021). Notably, comorbid depressive symptoms can further reduce treatment adherence and quality of life of patients, increasing the burden on patients, their families, and society. In this context, it is important to prioritize early screening of comorbid depressive symptoms in epilepsy patients and to implement prompt effective interventions targeting risk factors (Tsigebrhan et al., 2023; Lekoubou et al., 2019). Current research suggests that the pathogenesis of epilepsy with depressive symptoms may be a result of an interaction between multiple factors, including neurotransmitter imbalances, neuroinflammation, and abnormalities in the hypothalamic–pituitary–adrenal axis (Kanner, 2005; Shi et al., 2025; Kanner, 2009).

The gut microbiota can influence central nervous system (CNS) functioning through autonomic, endocrine, and immune pathways, and it is involved in the progression of neuropsychiatric disorders (Rieder et al., 2017). The oral microbiota, second to the gut microbiota, is the second-largest microbial community in human. Dysbiosis of the oral microbiota can affect brain function through the “oral-brain axis,” involving pathways including the trigeminal nerve, facial nerve, immune inflammation, and metabolic regulation (Bowland and Weyrich, 2022; Adil et al., 2025). Previous research identified an increase in α-diversity of the oral microbiota and a change in microbial community structure distinct from normal ones in patients with epilepsy (Lian et al., 2023). Additionally, the composition of the oral microbiota in patients with depression was also found to be different with that in healthy individuals, and the enrichment of certain differential microbial communities was associated with depressive state and symptom severity (Wingfield et al., 2021; Liu et al., 2025). Therefore, the oral microbiome may be associated with the onset and progression of neuropsychiatric disorders. However, there is currently a relative lack of research on the oral microbiota in epilepsy patients with depressive symptoms. In the present study, characteristics of the oral microbiota between epilepsy adults with and without depressive symptoms were compared by 16S rRNA gene sequencing technology, and related functional pathways were evaluated. This study aimed to identify potential key bacterial genera and pathways related to epilepsy with depressive symptoms, provides preliminary clues for further exploring the potential link between the “oral-brain axis” and epilepsy with depressive symptoms.

## 2 Experimental design

### 2.1 Research program

This study was approved by the Ethics Review Committee of the 940th Hospital of the Joint Logistics Support Force of the People’s Liberation Army (Approval No.: 2026KYLL084), and registered with the Chinese Clinical Trial Registry (Registration No.: ChiCTR2600121375). All samples and clinical data used in this study were collected with the consent of each participant. Tongue swab samples were collected from 59 adult patients who were diagnosed with epilepsy by a neurologist between April and May 2026. At the same time, data on the number of antiepileptic drugs used and lifestyle factors (e.g., smoking and oral hygiene habits) were collected as potential confounding factors for logistic regression analysis. Inclusion criteria: (1) age 18–65 years; (2) epilepsy was diagnosed and seizures were classified based on the 2017 International League Against Epilepsy (ILAE) Classification and Definitions of Epilepsy. Exclusion criteria: (1) use of antibiotics, probiotics, prebiotics, or supplements containing vitamins, protein, or unsaturated fatty acids within the past 3 months; (2) a diagnosis of oral disease within the past 3 months; (3) a history of autoimmune disease; (4) a history of or current malignant tumors, or a history of gastrointestinal surgery; (5) mental disorders other than epilepsy-related depressive symptoms, such as anxiety disorders, autism spectrum disorders, and schizophrenia; (6) presence of other metabolic conditions, such as hypertension, diabetes, obesity, and metabolic syndrome; (7) pregnant or breastfeeding females; (8) severe malnutrition, infections, or a history of substance abuse or alcoholism; (9) status epilepticus. The Neurological Disorders Depression Inventory for Epilepsy (NDDI-E) was applied to conduct an initial screening for depressive symptoms in patients with epilepsy. The NDDI-E was used solely as a screening tool for depressive symptoms and as a basis for grouping participants in this study. It does not constitute a clinical diagnosis of depressive disorder. The study participants were divided into two groups based on their NDDI-E scores: epilepsy with depressive symptoms (EDS, NDDI-E > 12, *n* = 28) and epilepsy (EP, NDDI-E ≤ 12, *n* = 31).

### 2.2 Tongue swab sampling

Tongue-coating samples were collected according to the method proposed by Lu et al. (2022). All participants underwent an oral health screening prior to sampling to rule out any medical conditions that might affect the composition of the oral microbiota. Sampling was completed in the early morning after admission, before teeth brushing and eating. After mouth rinsing, tongue-coating samples were collected from the dorsal surface of the tongue to the mid-body region using disposable sterile swabs. All samples were placed immediately in a sterile cryovial and stored at −80 °C for future use.

### 2.3 DNA extraction and polymerase chain reaction (PCR) amplification

Genomic DNA was extracted from the samples using the MagPure Soil DNA KF Kit (Magan) in accordance with the manufacturer’s instructions. The concentration and purity of DNA were determined using a NanoDrop 2000 (Thermo Fisher Scientific, USA) and agarose gel electrophoresis. Extracted DNA was stored at −20 °C. By using the extracted genomic DNA as a template, PCR amplification of the bacterial 16S rRNA gene was performed with barcoded specific primers and Takara Ex Taq high-fidelity polymerase. The V3-V4 variable regions of the 16S rRNA gene were amplified using the universal primers 343F (5’-TACGGRAGGCAGCAG-3′) and 798R (5’-AGGGTATCTAATCCT-3′) for oral microbial diversity analysis.

### 2.4 NovaSeq sequencing and sequence data processing

Shanghai Ouyi Biomedical Technology Co., Ltd. (China) completed library preparation, sequencing, data analysis, and gene sequencing. PCR products were sequenced on the Illumina NovaSeq 6,000 platform, and the raw data were stored in FASTQ format. Cutadapt software was used to trim the primer sequences from the raw data sequence. Sequence quality control, including quality filtering, denoising, paired-end merging, and chimera removal, was performed using DADA2 in QIIME2 with default parameters (November 2020). Tables were drawn to show the abundance of representative sequences and amplicon sequence variants (ASVs).

### 2.5 ASV clustering and taxonomy annotation

Representative sequences were aligned against databases for taxonomy annotation. 16S sequences were aligned using the Silva (version 138) database, and species-level annotation was performed using the q2-feature-classifier software with default parameters.

### 2.6 Bacterial diversity and taxonomic analysis

Alpha (α) and beta (β) diversity analyses were performed using QIIME2. The α-diversity was assessed using the Chao1, Shannon, and Simpson index. Principal Coordinate Analysis (PCoA) using R package revealed differences in the oral microbiota between different groups. With an uncorrected *p* < 0.05 serving as the screening criterion for differentially abundant genera; FDR-corrected values calculated using the Benjamini–Hochberg method are reported in the Supplementary Table S1. Comparison of the differences was inferred with Wilcoxon statistical algorithm implemented in R package. Linear discriminant analysis effect size (LEfSe) was used to reveal species with differential abundance between groups.

### 2.7 Kyoto encyclopedia of genes and genomes (KEGG) ortholog (KO) functional annotation

PICRUSt2 software was used to infer the KO composition of each ASV based on representative ASV sequences and abundance data obtained from 16S rRNA amplicon sequencing, through sequence alignment and hidden state prediction. After correcting for 16S rRNA gene copy numbers, the predicted KO functional abundance profiles at the sample level were created. It should be noted that the results obtained using PICRUSt2 reflect the potential functional composition of the microbial community as inferred from 16S rRNA sequences, rather than the actual gene abundance, gene expression levels, or host signaling pathway activity as measured by metagenomic analysis.

### 2.8 Statistical analysis

Statistical analyses were performed using SPSS 27.0 and R software (version 4.5.0). The normality of measurement data was assessed by Shapiro–Wilk normality test. Data that followed a normal distribution were expressed as mean ± standard deviation (x̄ ± s), and comparisons between groups were performed using an independent samples *t*-test; data that did not follow a normal distribution were expressed as median (interquartile range, IQR), and comparisons between groups were completed with Man-Whitney U test. Categorical data were presented as frequencies (%), and comparisons between groups were analyzed with chi-square test or Fisher’s exact test. Given the small sample size in this study (*n* = 59), a Firth-corrected logistic regression model was used for analysis to reduce small-sample bias and the effect of complete separation. Univariate Firth-corrected logistic regression analysis was performed to identify candidate factors related to epilepsy with depressive symptoms. Variables that were statistically significant (*p* < 0.05) in the univariate analysis were included in the multivariate analysis. Given that clinical and lifestyle factors, such as age, gender, use of antiepileptic drugs, frequency of toothbrushing, smoking status, seizure frequency, and seizure type, may simultaneously influence the manifestation of depressive symptoms and the composition of the oral microbiome in patients with epilepsy, these variables were further included as covariates in a Firth-corrected multivariate logistic regression model to adjust for potential confounding factors. Multicollinearity of the included variables was tested using variance inflation factor (VIF). VIF < 3 indicated no significant multicollinearity. Multivariate Firth-corrected logistic regression analysis was applied to assess independent clinical variables and differential bacterial genera related to epilepsy with depressive symptoms. All statistical tests were two-sided, and a *p*-value of less than 0.05 was considered statistically significant.

## 3 Results

### 3.1 Participant enrollment and baseline clinical characteristics

After rigorous screening, a total of 59 patients were enrolled (EDS, *n* = 28; EP, *n* = 31). The process and flowchart are provided in Figure 1. The median age was 27 years in the EDS group and 33 years in the EP group. The male to female ratio was 43%: 57 and 58%: 42% in the EDS and EP groups, respectively. There were no statistically significant differences between the two groups in terms of the use of antiepileptic drugs, frequency of toothbrushing, smoking, seizure frequency, or seizure type (all *p* > 0.05). Baseline clinical characteristics of the two groups are shown in Table 1.

**Figure 1:**
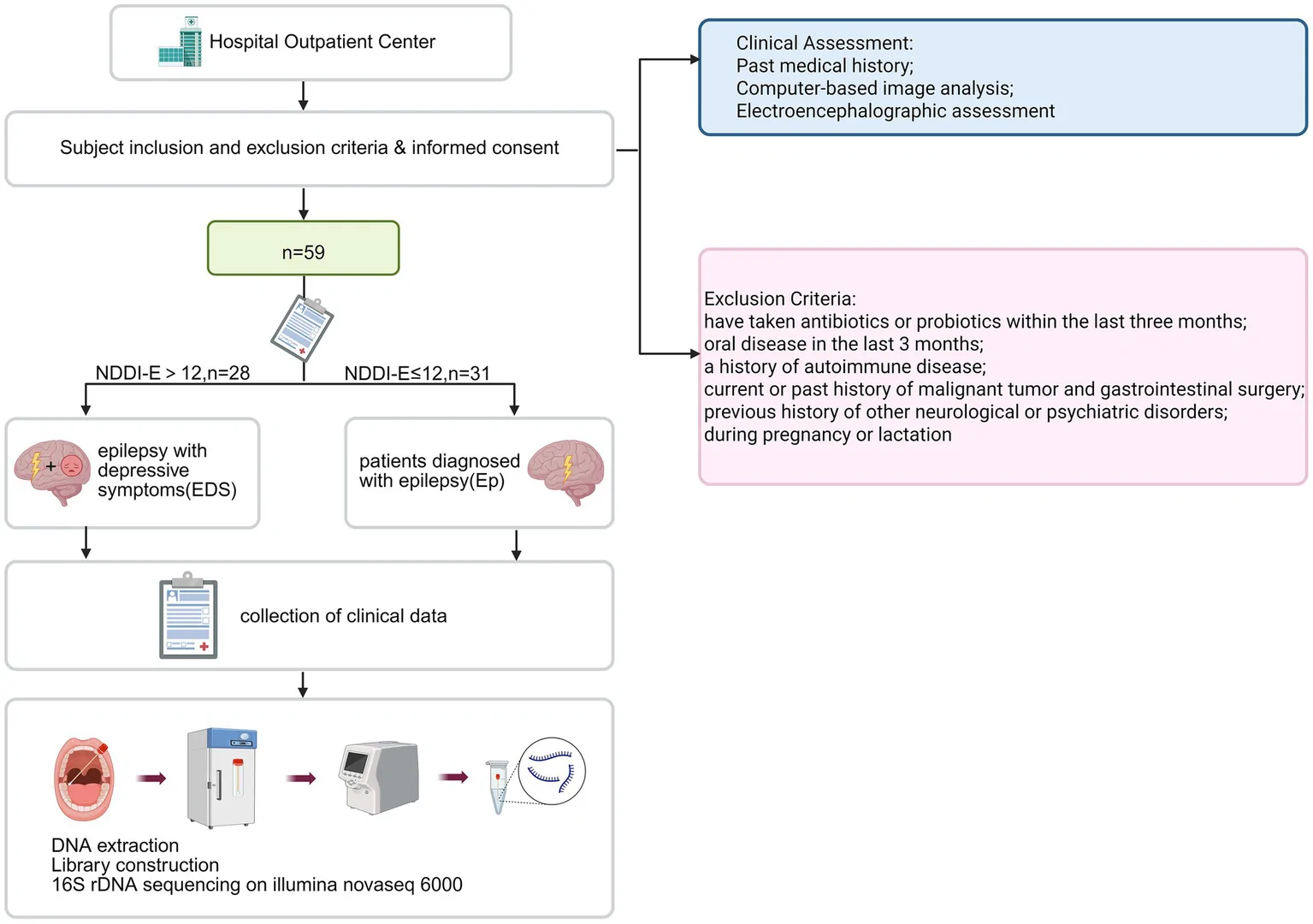
The flowchart.

**Table 1:** Characteristics of the participants in this study.

Characteristics	EDS (*N* = 28)	EP (*N* = 31)	*p*-value
Gender			0.301
Male, *n* (%)	12 (43%)	18 (58)	
Female, *n* (%)	16 (57%)	13 (42)	
Age (years)	27 (22, 32)	33 (22, 39)	0.096
Number of ASM (*n*, %)			0.104
0	2 (7)	7 (23)	
1	16 (57)	19 (61)	
≥2	10 (36)	5 (16)	
Toothbrushing frequency (per day) (*n*, %)			0.561
1	6 (21)	9 (29)	
≥2	22 (79)	22 (71)	
Smoking (*n*, %)			0.264
Yes	6 (21)	11 (35)	
No	22 (79)	20 (65)	
Frequency (seizure per month) (*n*, %)			>0.99
≤1	20 (71)	22 (71)	
>1	8 (29)	9 (29)	
Seizure type (*n*, %)			0.095
Focal seizures	20 (71)	28 (90)	
Generalized seizures	8 (29)	3 (10)	

EDS, epilepsy with depressive symptoms; EP, epilepsy; ASM, Antiseizure Medications.

### 3.2 Oral microbiological characteristics

#### 3.2.1 Sequencing depth and microbial diversity analysis

Dilution curve analysis showed that the whole-tree phylogenetic diversity (PD) index for each sample gradually leveled off as sequencing depth increased, suggesting that the sequencing depth used in this study was sufficient to fully capture the microbial diversity from the samples (Figure 2A). Analysis of α-diversity indicated slightly higher richness and evenness of the oral microbial communities in the EDS group than in the EP group (Figures 2B–D). However, the differences between the two groups were not statistically significant as evidenced by the Chao-1 index (*p* = 0.2), Shannon index (*p* = 0.47), and Simpson index (*p* = 0.83). The β-diversity analysis results were represented in a PCoA plot (Figure 2E), and both two groups showed some tendency to diverge. Additionally, there was no significant difference in binary Jaccard distance (R = 0.0192, *p* = 0.093, Adonis), suggesting that the composition of the oral microbial communities was similar between the two groups. The Venn diagram (Figure 2F) identified 848 ASVs shared by EDS (*n* = 1,202) and EP (*n* = 1,117).

**Figure 2:**
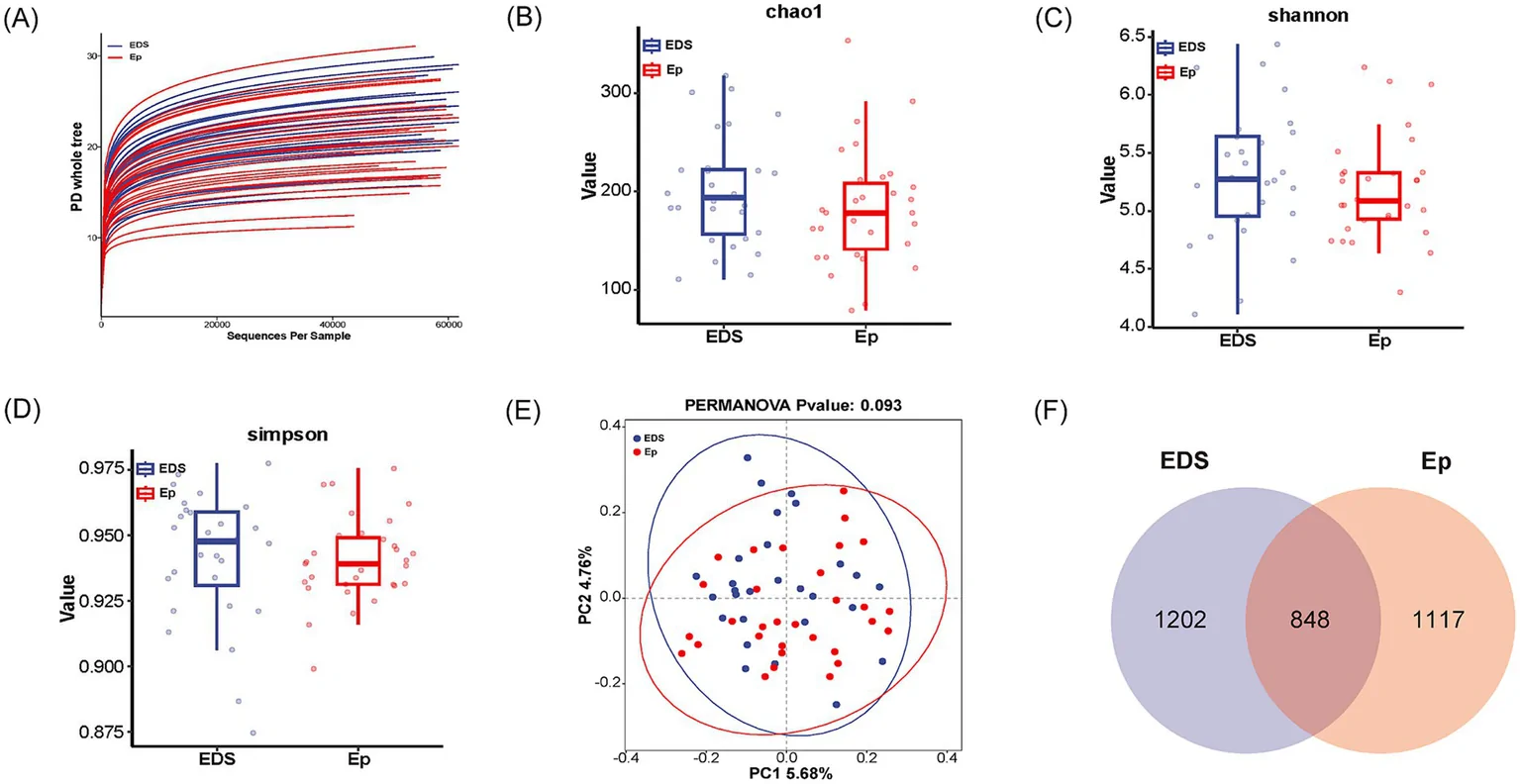
Differences between EDS and EP in terms of α-diversity and β-diversity of oral microbiota. **(A)** The PD_−_whole_−_tree analysis indicates that as the sequencing depth increases, the microbial diversity indices for each sample do not increase significantly, suggesting that the sequencing depth is sufficient. **(B–D)** The Chao1 index, Shannon index, and Simpson index indicate that the richness and diversity of the oral microbiota in the EDS group were slightly higher than those in the EP group. **(E)** Binary-Jaccard PCoA (PC1–PC3) analysis revealed no significant differences in community structure between EDS and EP. **(F)** The Venn diagram shows the number of ASVs shared by EDS and EP, as well as the number of ASVs unique to each. ASV, Amplicon sequence variant; PCoA, Principal coordinate analysis; EDS, Epilepsy with depressive symptoms; EP, Epilepsy.

#### 3.2.2 Oral microbial community composition and differential genera

At the phylum level (Figure 3A), the overall microbial community composition was similar between the two groups, without significant structural differences. The dominant phyla in both groups were *Firmicutes*, *Bacteroidota*, *Actinobacteriota*, *Proteobacteria*, *Fusobacteriota, Campylobacterota*. At the genus level (Figure 3B), the dominant genera in both groups were *Streptococcus*, *Prevotella*, *Actinomyces*, *Neisseria*, *Haemophilus*, suggesting that the overall microbial community structure was consistent between the two groups. Based on the Wilcoxon rank-sum test, differential abundance analysis at the genus level was further performed using an unadjusted *p* value < 0.05 as the screening criterion (Figure 3C). The results showed that *Bacillus*, *Fusicatibacter*, and *[Eubacterium]_coprostanoligenes_group* were significantly enriched in EP; while *Dialister*, *Olsenella* were significantly enriched in EDS. Notably, *Cryptobacterium*, *Pseudoramibacter*, *Thermomonas* were only detected in EDS.

**Figure 3:**
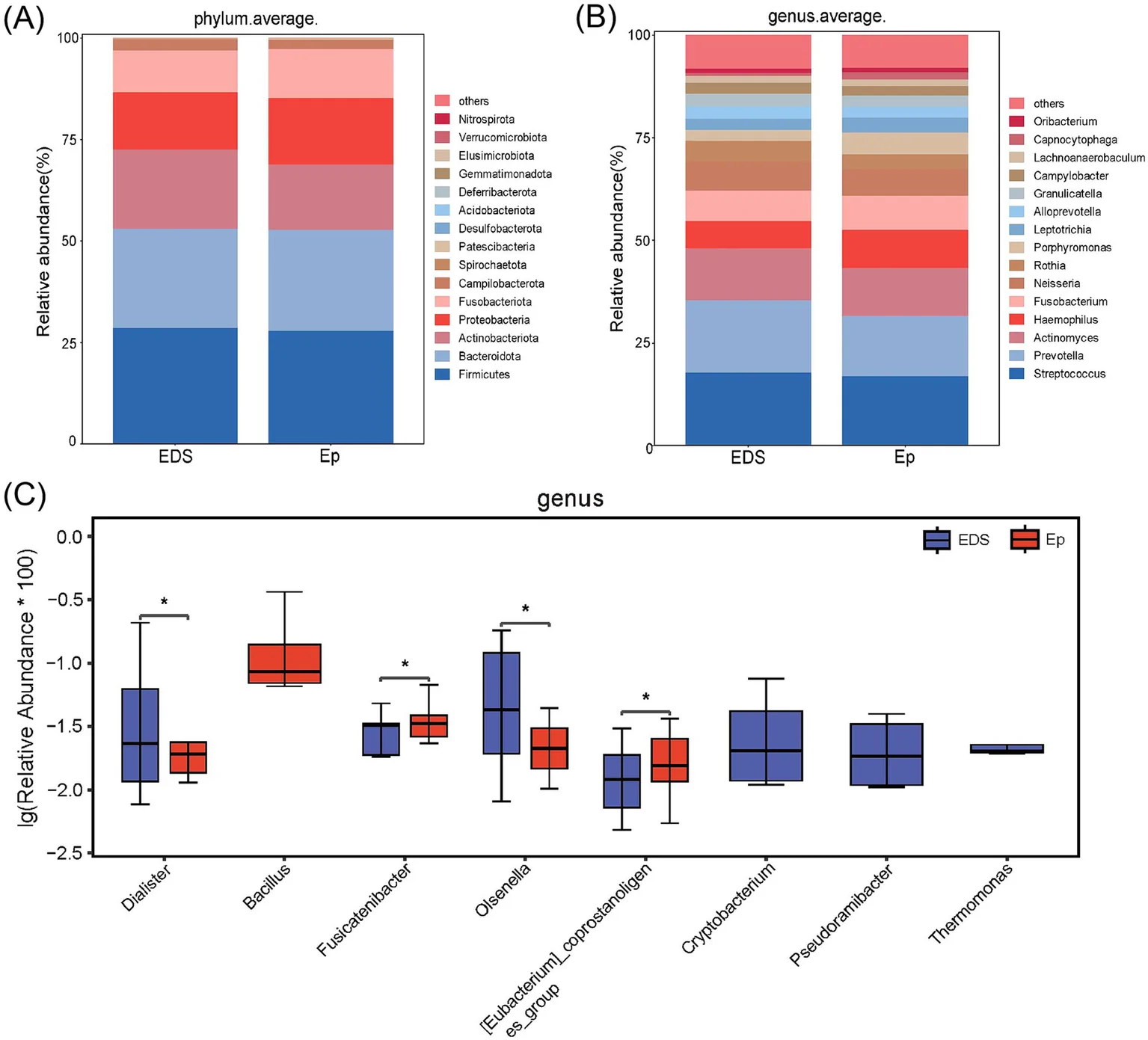
Composition and differences in oral microbiota of EDS and EP at the phylum and genus levels. **(A)** At the phylum level, EDS and EP represent the distribution of species composition. **(B)** At the genus level, the average species composition of EDS and EP is relatively uniform. **(C)** At the genus level, there are significant differences in oral microbiota between EDS and EP. EDS, epilepsy with depressive symptoms; EP, epilepsy.

#### 3.2.3 Key differential genera and functional pathways

LEfSe analysis was applied to identify key bacterial genera with discriminatory power. It was noted that *Bacillus* was significantly enriched in the EP group, while *Dialister* was highly enriched in the EDS group. *Bacillus* and *Dialister* may serve as potential marker bacterial genera for distinguishing between EP and EDS (Figure 4A). To explore the potential functional characteristics associated with microbial differences, PICRUSt2 was applied for functional prediction analysis. The results showed that the predicted abundance of functions annotated to the RIG-I-like receptor signaling pathway was higher in the EDS group than in the EP group (*p* = 2.2 × 10^−2^, Figure 4B).

**Figure 4:**
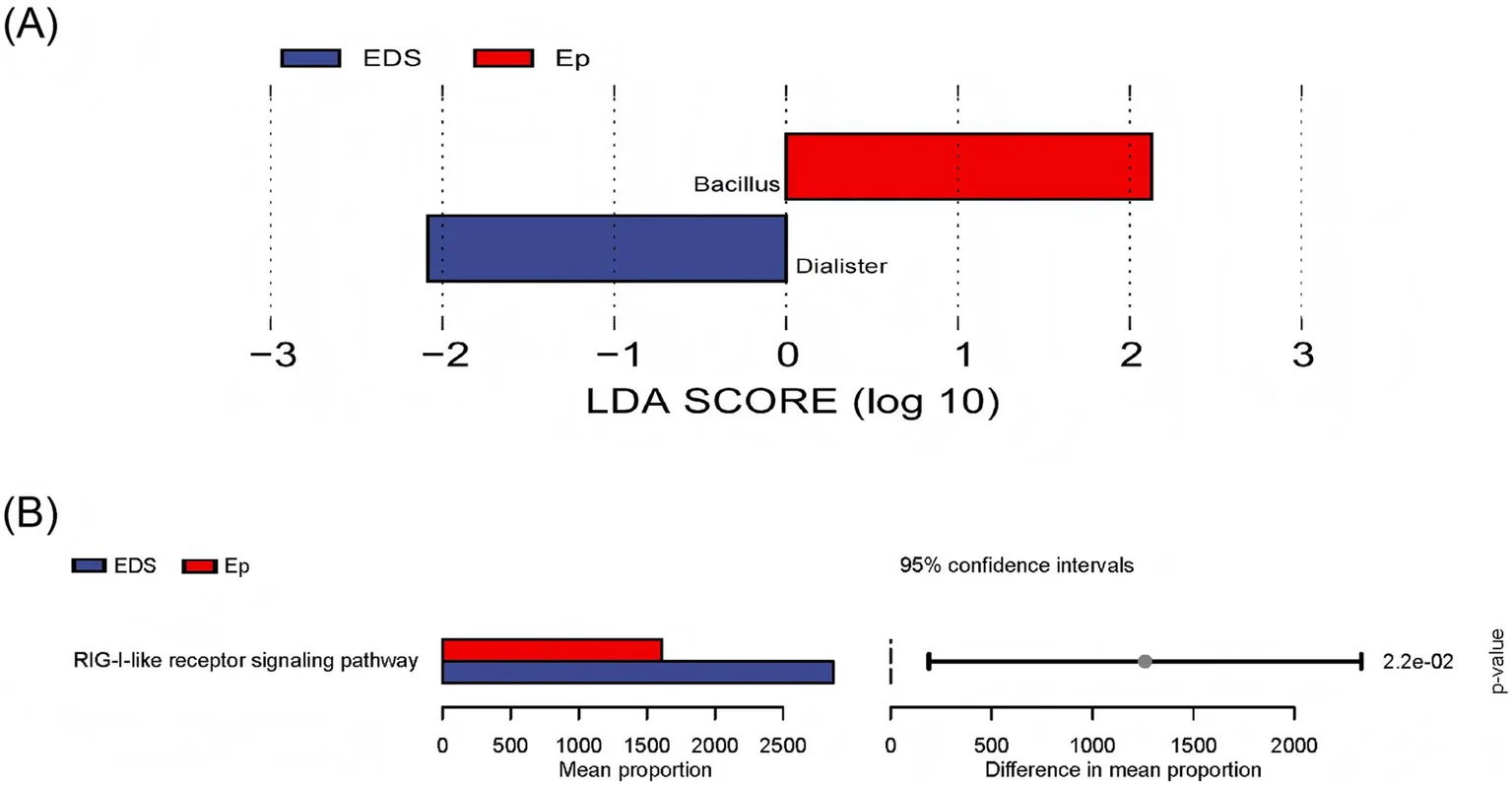
Analysis of key differential bacterial genera associated with EDS and predicted functional pathways. **(A)** The histogram of LDA value distribution shows the microorganisms with a significant difference at the genus level between the two groups (*p* < 0.05, LDA > 2). **(B)** Based on the PICRUSt2-predicted functional profiles, the predicted functional abundance annotated to the RIG-I-like receptor signaling pathway was higher in the EDS group than in the EP group (*p* = 0.022). EDS, epilepsy with depressive symptoms; EP, epilepsy.

#### 3.2.4 Firth-corrected logistic regression analysis of factors associated with EDS

Univariate Firth-corrected logistic regression analysis results (Table 2) revealed that use of ≥ 2 types of antiepileptic drugs (OR = 5.727, 95% CI: 1.077–40.193, *p* = 0.040), *Dialister* (OR = 1.651, 95% CI: 1.082–2.631, *p* = 0.019), and *Bacillus* (OR = 0.437, 95% CI: < 0.001–0.869, *p* = 0.012) were significantly associated with EDS. Clinically relevant variables identified in the univariate analysis were included in a multivariate Firth-corrected logistic regression model (Table 3). After adjusting for age, sex, number of antiepileptic drugs used, toothbrushing frequency, smoking status, seizure frequency, seizure type, and candidate bacterial genera, only *Dialister* abundance was found to be statistically associated with EDS (OR = 1.646, 95% CI: 1.007–2.896, *p* = 0.047). The effect sizes and their 95% CIs of each variable are displayed in a Forest plot (Figure 5). It was noted that the 95% CI for *Dialister* did not cross 1, consistent with the regression analysis result. However, the 95% CIs for some variables were relatively wide, suggesting a degree of uncertainty in the estimates. Thus, the association between *Dialister* and EDS requires further validation in studies with larger sample sizes and independent research.

**Table 2:** Univariate Firth logistic regression analysis of the association between various variables and depressive symptoms in patients with epilepsy.

Characteristics	β	SE	OR	95% CI	*p*-value
Age	−0.035	0.024	0.966	0.917–1.011	0.136
Gender (Female)	0.593	0.518	1.809	0.660–5.089	0.250
Number of ASM					
=1	0.932	0.803	2.538	0.577–15.087	0.226
≥2	1.745	0.900	5.727	1.077–40.193	0.040*
Toothbrushing frequency (≥2/day)	0.379	0.590	1.462	0.464–4.828	0.518
Smoking (No)	0.664	0.578	1.942	0.638–6.311	0.245
Seizure frequency (>1/month)	−0.018	0.562	0.982	0.321–2.973	0.974
Seizure type (Generalized seizures)	1.217	0.698	3.376	0.913–15.112	0.069
*Dialister*	0.501	0.224	1.651	1.082–2.631	0.019*
*Bacillus*	−0.828	0.478	0.437	<0.001–0.869	0.012*

ASM, antiseizure medication.

**Table 3:** Multivariate Firth-corrected logistic regression analysis of the association between various variables and depressive symptoms in patients with epilepsy.

Characteristics	β	SE	OR	95% CI	*p*-value
Age	−0.019	0.027	0.981	0.922–1.036	0.496
Gender (Female)	1.002	0.700	2.724	0.680–12.161	0.158
Number of ASM					
=1	1.363	0.914	3.907	0.655–31.428	0.138
≥2	1.863	1.032	6.444	0.855–66.638	0.071
Seizure frequency (>1/month)	−0.015	0.716	0.985	0.222–4.567	0.985
Toothbrushing frequency (≥2/day)	0.627	0.733	1.873	0.434–9.738	0.408
Smoking (No)	−0.036	0.760	0.964	0.199–4.634	0.963
Seizure type (Generalized seizures)	1.025	0.841	2.787	0.543–18.439	0.225
*Dialister*	0.498	0.255	1.646	1.007–2.896	0.047*
*Bacillus*	−0.462	0.379	0.630	0.129–1.208	0.186

ASM, antiseizure medication.

**Figure 5:**
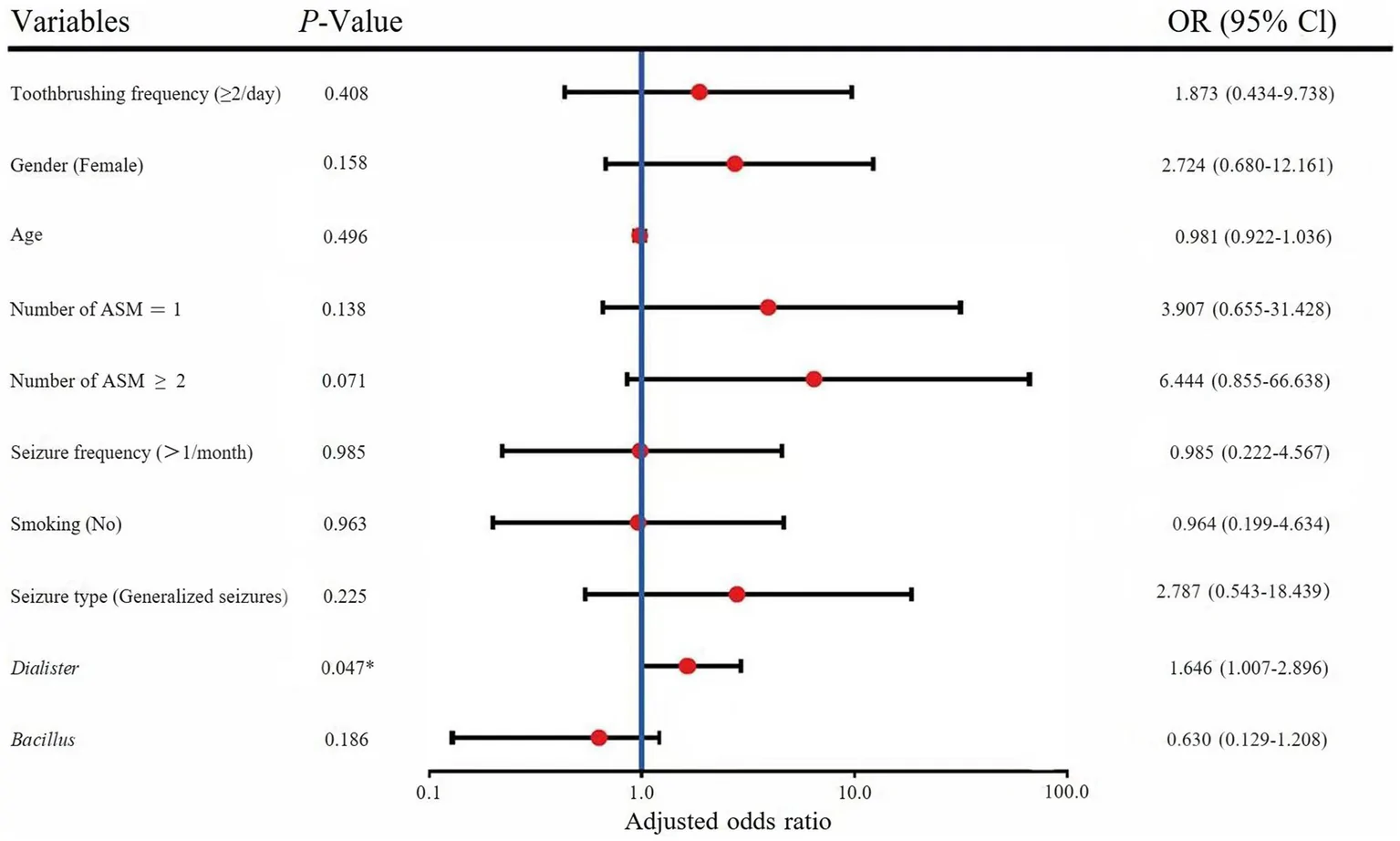
Forest plot of Firth-corrected multivariate logistic regression analysis. Shows the strength of the association between each factor and EDS, expressed as odds ratios (OR) and their 95% confidence intervals. ASM, antiseizure medication.

## 4 Discussion

In this study, a comparison of the oral microbiota between epilepsy patients with and without depressive symptoms was performed using 16S rRNA sequencing. No significant differences were observed between the two groups in terms of overall microbial community composition or diversity. At the genus level, *Dialister* exhibited a higher relative abundance in patients with epilepsy and depressive symptoms, while *Bacillus* had a higher relative abundance in patients with epilepsy only. Previous studies have compared patients with epilepsy or depression with healthy individuals. Lian et al. (2023) found that the richness and evenness of the oral microbiota in patients with epilepsy were both higher than those in healthy individuals, and that there were significant differences in microbial community composition. Research on depression (Wingfield et al., 2021) revealed no significant differences in α-diversity but a clear divergence in β-diversity between patients with depression and healthy individuals. In the present study, the relationship between oral microbiota and depressive symptoms in epilepsy patients was investigated. No significant differences were noted in the overall microbial community composition or diversity between the two groups, which may be related to the fact that the study population shared a common history of epilepsy. Compared to the overall remodeling of the oral microbiota, the co-occurrence of depressive symptoms may be more closely associated with changes in the relative abundance of specific bacterial genera.

Further analysis of the differential bacterial genera revealed that *Bacillus* was detected only in patients with epilepsy. *Bacillus* is a genus of Gram-positive bacteria primarily found in soil, air, and fermented foods. In the oral microbiota, *Bacillus* typically appears as a transient colonizer with low abundance (Khan et al., 2023). Furthermore, its association with EDS was no longer observed in the multivariate Firth-corrected logistic regression analysis of the present study. It remains to be tested that *Bacillus* has a direct protective or pathogenic effect on patients with epilepsy with depressive symptoms. In contrast, *Dialister* showed an increased relative abundance in patients with epilepsy and depressive symptoms and remained significantly correlated in multivariate analysis. *Dialister* may be a potential marker bacterium for epilepsy with depressive symptoms, and its potential biological significance warrants further exploration.

*Dialister* is a Gram-negative anaerobic bacterium (Morio et al., 2007) that typically exists as a low-abundance commensal bacterium in healthy individuals. Abundance of *Dialister* can change significantly under specific physiological or pathological conditions (Mena-Vázquez et al., 2023). In addition, *Dialister* can be found simultaneously in the host’s oral and gut microbiota, suggesting that it may have the ability to colonize across these regions (Costa et al., 2024). Previous studies on *Dialister* have primarily focused on the gut microbiota, and the patterns of change vary across different disease contexts. For example, abundance of the gut bacterium *Dialister* was found to increase significantly in patients with rheumatoid arthritis and obesity, which was independently associated with fat mass index (FMI). This may be due to the lipopolysaccharide-mediated increase in intestinal permeability and chronic inflammatory responses (Mena-Vázquez et al., 2023). In contrast, a significant reduction in the abundance of the gut bacterium *Dialister* was detected in patients with major depressive disorder, and *Dialister* was nearly depleted during periods of active symptoms (Caso et al., 2021). *Dialister* species found in the oral cavity are potentially pathogenic. For instance, *D. pneumosintes*, a key component of dental plaque biofilms, can participate in the pathological processes associated with gingivitis and periodontitis through virulence factors (Varshan and Sankar, 2024). Periodontitis is not merely a localized oral disease, and it is closely linked to systemic inflammation. Research by Luthra *et al.* found that treatment of periodontitis lowered serum C-reactive protein (CRP) levels, suggesting that periodontitis may be a potential trigger of systemic inflammation (Luthra et al., 2023). In all, the enrichment of oral *Dialister* in patients with epilepsy and depressive symptoms may not only reflect a change in oral microbial composition but also suggest a potential association with local oral inflammation, as well as a potential link to systemic inflammatory processes and neuropsychiatric conditions. This is consistent with the “oral-brain axis” theory, which posits that there is bidirectional regulation of brain function by oral microbiota through immune, inflammatory, and neural signaling pathways (Adil et al., 2025).

Functional prediction analysis of the oral microbiota was devised to further investigate the host’s immune mechanisms potentially involved in the aforementioned changes in the microbiota. It was noted that the predicted abundance of the RIG-I-like receptor signaling pathway (RLR signaling pathway) was in EDS was significantly higher than in EP, suggesting that the oral microbiota associated with EDS may possess potential functional characteristics related to this pathway; however, this result does not indicate actual activation of the host’s RIG-I-like receptor signaling pathway.

RIG-I-like receptors (RLRs), a class of important intracellular pattern recognition receptors, primarily recognize nucleic acid molecules derived from viruses or bacteria. They activate downstream signaling cascades via the mitochondrial antiviral-signaling protein, thereby activating inflammatory transcription factors (e.g., NF-κB and IRF3/IRF7) and inducing the expression of type I interferons and various pro-inflammatory factors (Onomoto et al., 2021; Kronborg Hansen et al., 2022). Recent research has proved that the innate immune response mediated by the RLR signaling pathway is not only involved in antiviral immunity but may also be closely associated with inflammatory diseases of the CNS (Brand 3rd et al., 2015). From a microbiological perspective, nucleic acids of microbial origin and other microbe-associated molecular patterns (MAMPs) can be recognized by the host’s pattern recognition receptors, thereby influencing local and systemic immune responses (Chu and Mazmanian, 2013; Tan et al., 2018). Based on this biological context, we propose a hypothesis that requires further validation: EDS-associated changes in the oral microbiome may be related to the host’s innate immune recognition process. However, microbe-derived immunostimulatory molecules, RLR-associated molecules, and inflammatory markers were not evaluated in this study, precluding the determination of whether a direct association exists between Dialister and RLR signaling.

Neuroinflammation represents a critical pathological link between epileptic seizures and depressive symptoms. Altered glial cell function and neuroimmune dysregulation may both be associated with the onset of EDS (Wen et al., 2024). Therefore, the potential role of the RLR signaling pathway in EDS must be interpreted in the context of the central innate immune response. Inflammatory responses in the central nervous system involve diverse cell populations. Microglia, as the major resident innate immune cells of the brain, express pattern recognition receptors including RIG-I and MDA5 and can activate innate immune responses through MAVS-dependent signaling. Collectively, these observations raise the possibility that the RLR–MAVS signaling axis may contribute to the modulation of microglia-mediated neuroinflammatory processes (O'Brien et al., 2022; Cheng et al., 2017; Gern et al., 2024). Both epilepsy and depressive symptoms are accompanied by alterations in microglial states and chronic neuroinflammatory processes, suggesting that microglia may serve as an important regulatory node linking the pathological processes of these two conditions. During a seizure, the activation of microglia promotes the release of pro-inflammatory factors and increases neuronal excitability (Anaclerio et al., 2024; Li et al., 2023); it can also disrupt the balance between excitatory and inhibitory neurotransmission through abnormal synaptic remodeling, thereby increasing susceptibility to seizures (Wyatt et al., 2017; Wyatt-Johnson and Brewster, 2020; Stevens et al., 2007). During the development of depression, chronic stress, peripheral inflammation, and dysregulation of stress hormones may promote the transition of microglia from a homeostatic state toward an inflammation-associated functional state, accompanied by enhanced release of pro-inflammatory mediators. These microglial alterations may participate in depressive pathophysiology by disrupting neurotrophic signaling, suppressing hippocampal neurogenesis, and impairing synaptic plasticity and emotion-related neural circuit function (Yirmiya et al., 2015; Wang et al., 2022; Afridi and Suk, 2023). Based on the findings of the present study and existing evidence, EDS-associated alterations in the oral microbiota may be accompanied by changes in the functional characteristics of the RLR signaling pathway and may be potentially linked to neuroinflammatory processes. Given that the RLR–MAVS signaling axis is involved in microglia-mediated innate immune responses, microglia may represent a candidate cell type of interest for subsequent mechanistic investigations. However, the aforementioned association, as well as its underlying mechanisms and causal relationships, require further validation.

This study still has certain limitations. First, this was a single-center study with a relatively small sample size. Although the PD_−_whole_−_tree rarefaction curve tended to reach a plateau, indicating adequate sequencing depth for the included samples, this level of sequencing coverage cannot overcome the statistical limitations associated with the limited number of participants. The limited sample size may have reduced the statistical power to identify subtle differences between groups. Therefore, the lack of statistically significant differences in α- and β-diversity should not be interpreted as evidence that the microbial communities are completely identical between the two groups. Meanwhile, the magnitude of the association and its robustness between *Dialister* and depressive symptoms should also be interpreted with caution. Although Firth’s bias-corrected logistic regression was applied to reduce small-sample bias, these findings still require further validation in larger, multicenter, and independent cohorts. Second, the depressive symptoms in epilepsy patients in this study were assessed by neurologists, lacking a more strict diagnosis by psychiatrists. Additionally, the depressive symptoms in some of the enrolled patients may have been relatively mild. Thus, generalizability of the study’s findings to the general population with major depressive disorder has some limitations. Although this study adjusted for smoking status, toothbrushing frequency, and the number of antiseizure medications used, and excluded participants with recently diagnosed oral diseases or those who had received interventions that might affect the oral microbiota at enrollment, residual confounding factors cannot be completely ruled out. Dietary patterns, periodontal status, medication types, and duration of medication use may all influence oral microbiota composition; however, these factors were not systematically quantified or adequately adjusted for in the present study. Therefore, these findings require further validation in prospective cohort studies. Third, 16S rRNA sequencing combined with PICRUSt2 functional prediction only provides preliminary insights into the oral microbial characteristics and potential functional alterations associated with depressive symptoms in epilepsy patients. Further studies combining metagenomic, metabolomic, and immunological/inflammatory markers are needed to validate the biological significance of relevant microbial communities and functional pathways.

## 5 Conclusion

This exploratory study utilizing 16S rRNA sequencing reveals compositional variations in the oral microbiome correlated with epilepsy and comorbid depressive symptoms. Significantly, the genus *Dialister* exhibited an enrichment pattern in patients with epilepsy and comorbid depressive symptoms and remained independently associated with depressive symptoms after adjustment for potential confounding factors, suggesting its potential as a candidate microbial biomarker for this clinical phenotype. Functional prediction analysis further indicated that shifts in the oral microbiome related to depressive symptoms may be accompanied by changes in innate immune-related functional characteristics, particularly involving the RIG-I-like receptor signaling pathway. Considering the potential role of this pathway in neuroimmune regulation, microglia may emerge as a promising candidate cell type for further mechanistic studies. These findings provide preliminary insights into the potential role of the oral–brain axis in epilepsy with comorbid depressive symptoms.

## Data Availability

The data presented in the study are deposited in the NCBI database repository, BioProject accession number PRJNA1515868 and SRA accession SRP728766.

## Glossary

EP: Epilepsy
EDS: Epilepsy with Depressive Symptoms
16S rRNA: 16S ribosomal RNA
ASV: Amplicon Sequence Variant
CI: Confidence Interval
CNS: Central Nervous System
CRP: C-reactive Protein
DADA2: Divisive Amplicon Denoising Algorithm 2
DNA: Deoxyribonucleic Acid
ILAE: International League Against Epilepsy
IQR: Interquartile Range
KEGG: Kyoto Encyclopedia of Genes and Genomes
KO: KEGG Ortholog
LEfSe: Linear Discriminant Analysis Effect Size
NDDI-E: Neurological Disorders Depression Inventory for Epilepsy
OR: Odds Ratio
PCoA: Principal Coordinate Analysis
PCR: Polymerase Chain Reaction
PD: Phylogenetic Diversity
QIIME2: Quantitative Insights Into Microbial Ecology 2
RIG-I: Retinoic Acid-inducible Gene I
RLR: RIG-I-like Receptor
SPSS: Statistical Package for the Social Sciences
VIF: Variance Inflation Factor
